# Role of ‘dusting and pop-dusting’ using a high-powered (100 W) laser machine in the treatment of large stones (≥ 15 mm): prospective outcomes over 16 months

**DOI:** 10.1007/s00240-018-1076-4

**Published:** 2018-08-21

**Authors:** Amelia Pietropaolo, Patrick Jones, Lily Whitehurst, Bhaskar K. Somani

**Affiliations:** 1 Department of Urology, University Hospital Southampton NHS Trust, Southampton, SO16 6YD UK; 2 University of Southampton, Southampton, UK

**Keywords:** Laser, Stone, Fragmentation, Ureteroscopy, Dusting, Popcorn, Pop-dusting

## Abstract

Ureteroscopy and laser stone fragmentation (URSL) has had recent advancements with the more powerful laser systems with the ability to ‘dust’ and ‘pop-dust’ the stone. We wanted to look at the outcomes of this method for large stones (≥ 15 mm) using our new 100 W holmium laser. Over a period of 16 months (January 2017–April 2018), 50 patients underwent URSL for minimum cumulative stone size of ≥ 15 mm. Data were collected prospectively on patient and stone demographics and outcomes of URSL. The laser setting used was a power of 0.3–0.6 J and a frequency of 20–50 Hz using a long-pulse setting with a 272-µm fiber. Fifty patients underwent 55 URSL procedures (5 bilateral procedures) using dusting and pop-dusting settings. The mean age was 58 years (range 2–88 years) with a male:female ratio of 35:15. The mean single and overall stone size were 10.3 mm (3–23 mm) and 21 mm (range 15–52 mm) with two-thirds of all patients (65%) having multiple stones. The stone location was in the kidney (*n* = 65, 78%), in the ureter (*n* = 19, 22%) and 5 patients had bilateral renal stones. With a mean operating time of 51 min, the initial and final SFR were 93 and 98%, respectively. A pre-operative stent, access sheath and a post-operative stent were present in 29 (53%), 34 (62%) and 51 (93%) procedures, respectively. Over a mean hospital stay of 0.6 days (74% day-case procedures), there was one Clavien IV complication related to urosepsis but without any other major or minor complications. Dusting and pop-dusting techniques achieve an excellent SFR with low risk of complications even for large stones. This might set a new benchmark for treating large stones, bilateral or multiple stones in a single setting, without the need for secondary procedures in most cases.

## Introduction

Over the last two decades, there has been an ascent of ureteroscopy for treatment of kidney stone disease (KSD) [1]. The use of laser lithotripsy using holmium:yttrium–aluminum–garnet (Ho:YAG) laser allows for fragmentation and retrieval of stones. Other treatment methods that are gaining more popularity include dusting, popcorning and pop-dusting [2, 3]. The advantage of dusting seems to be a reduction in operative time, ureteral access sheath (UAS) usage, fragment retrieval and complications related to basketing them [3, 4].

The high-power, high-frequency, long-pulse Ho:YAG lasers allow for dusting and pop-dusting, producing dust which can evacuate spontaneously. The first stage starts with dusting using a low energy (0.2–0.5 J), high frequency (40–50 Hz) and long pulse length in a contact mode. If the stone is hard, then the second stage (completion) allows pop-dusting using low pulse energy (0.5–0.6 J), high frequency (20–40 Hz) and long pulse mode in a non-contact lithotripsy to pulverize the stone [2].

Recently, there has been a rise in the newer forms of dusting techniques [4–6]. While the studies have shown them to be effective for small to medium size stones, the efficacy of combined dusting and pop-dusting for larger size stones still remains unclear. With the increasing use of dusting and pop-dusting techniques we wanted to see the results of ureteroscopy and laser stone fragmentation (URSL) using these techniques for outcomes of larger stones (≥ 15 mm).

## Materials and methods

Over a period of 16 months (January 2017–April 2018), consecutive patients with stones ≥ 15 mm treated with dusting and pop-dusting were included in our study. Our ureteroscopy outcome audit was registered with our hospital ‘Clinical Effectiveness and Audit’ department with patient consent for participation obtained prior to the procedure. Outcomes were collated prospectively and recorded in our prospective database which was then analyzed for patient demographics, stone parameters, pre-operative assessment, operative details, length of stay (LoS), stone-free rate (SFR) and complication rates.

A 100-W high-power Ho:YAG system (Lumenis, Inc.) was used. The energy setting used varied between 0.2 and 0.6 J with a frequency of 20–50 Hz giving a total power of 4–30 W. We started with the dusting setting [(0.2–0.5 J), (40–50 Hz)] and switched to pop-dusting [(0.5–0.6 J), (20–40 Hz)] for the completion of procedure. A reusable 272-µm laser fiber (Lumenis, Inc.) was used for all cases irrespective of the stone size or location and the tip was cut with simple scissors to ‘renew the tip’ [2].

Using our standard ureteroscopy technique and a day-case protocol as described previously [7], patients were planned for discharge on the same day or the following morning. The flexible ureteroscopy was done using the Storz flex X2, with an access sheath (9.5 F/11.5 F or 12 F/14 F Cook Flexor sheath) used if appropriate. SFR was defined as 2U or 2X [8], having complete absence of stones endoscopically or clinically insignificant fragments ≤ 2 mm on USS or XR KUB. Post-operative complications were recorded as per the Clavien–Dindo grading [9]. A 6-F post-operative stent was placed post-URS and this was subsequently removed 1–3 weeks post-procedure. Peri-operative antibiotics were given as per the urine culture results or as per the departmental protocol and routine post-operative urethral catheter was not placed. When possible, an attempt was made to remove a single stone fragment for stone analysis using a Cook Ngage stone extractor (Cook Medical, USA).

## Results

Fifty patients underwent 55 ureteroscopy and laser stone fragmentation (URSL) procedures (5 bilateral procedures) using dusting and pop-dusting settings (Table 1). The mean age was 58 years (range 2–88 years) with a male:female ratio of 35:15. The mean single and overall stone size was 10.3 mm (3–23 mm) and 21 mm (range 15–52 mm) with two-thirds of all patients (65%) having multiple stones. The stone location was in the kidney (*n* = 65, 78%), in the ureter (*n* = 19, 22%) and 5 patients with bilateral renal stones underwent bilateral simultaneous URSL procedures.

**Table 1:** Patient demographics and overall outcomes of the study

Patient demographics and clinical data (patients *N* = 50; renal units *N* = 55)	*n* (%) ± SD
Male:female (*n*)	35:15
Mean age, years (range)	58 (2–88)
Stone size (cumulative) in mm	21 (range 15–52 mm)
*Stone location*	
Lower calyx	34
Renal pelvis	15
Upper calyx	11
Middle calyx	5
Pelvi-ureteric junction	4
Ureter	15
(Multiple bilateral kidney stones)	(36)
(Multiple kidney + ureter)	(19)
*Number of renal units* (55)	
Access sheath (%)	(62%)
Size	
9.5/11.5	6
12/14	26
14/16	2
Pre-operative stent, *n* (%)	25 (45%)
Post-operative stent placement, *n* (%)	51 (93%)
Mean hospital stay (range)	0.6 days (0–7)
Complications	1 (Clavien IV) (Urosepsis, ICU admission)
Stone-free rate, *n* (%)	Initial 93% Final 98% (1.1 ± 0.27 procedure per patient)
*Stone composition*	
Calcium oxalate monohydrate	11
Calcium phosphate carbonate	2
Calcium oxalate dihydrate + calcium oxalate monohydrate	4
Magnesium ammonium phosphate hexahydrate + calcium phosphate carbonate	7
Calcium oxalate monohydrate + calcium phosphate carbonate	7
Calcium oxalate dihydrate + calcium oxalate monohydrate + calcium phosphate carbonate	5
Calcium hydrogen phosphate dihydrate	1
Magnesium ammonium phosphate	3
Cystine	3
Uric acid	7

With a mean operating time of 51 min (range 15–100 min), the SFR (across 55 renal units) was 93% after the first procedure rising to 98% after the second procedure (needed in 3 patients). Of the patients who required a second procedure, one patient had a partial staghorn stone, one had a large stone in a buried diverticulum and the third had bilateral large stone burden. A pre-operative ureteric stent was present in 25 (46%) patients, a percutaneous nephrostomy (PCN) in 1 (2%) with no stent present in 29 (53%) patients. An access sheath was used in 34 (62%) procedures, with the size being 9.5 Fr/11.5 Fr (*n* = 6), 12 Fr/14 Fr (*n* = 26) and 14 Fr/16 Fr (*n* = 2). A post-operative ureteral stent was inserted in 51 (93%) procedures, removed 1–2 weeks after the procedure.

The mean hospital stay was 0.6 days (range 0–7 days), with 37/50 (74%) patients discharged the same day (day-case procedure). There was one major Clavien IV complication where a patient with a history of urosepsis required a brief ICU admission post-operatively but was subsequently discharged home. There were no other minor or major complications. Stone analysis was available in all 50 patients (Table 1).

## Discussion

### Meaning of the study

With a rise in the use of laser lithotripsy, our study shows a successful use of dusting and pop-dusting methods in consecutive patients with large stones (≥ 15 mm). The SFR (across 54 renal units) was 87% after the first procedure rising to 92% after the second procedure (needed in 3 patients). There was one Clavien IV complication wherein a patient with a previous history of urosepsis had a brief ICU admission post-URS due to sepsis but was subsequently discharged home. There were no other major or minor complications. The hospital stay was short with 74% patients discharged the same day.

### Comparison with other studies using the dusting techniques

Previous study from Michigan [4] showed good outcomes with dusting technique although they used a higher powered laser (120 W) compared to our study. Their access sheath use and SFR were lower and perhaps having an access sheath allows for better drainage and helps to clear out the dust thereby increasing the SFR, which might be more relevant for larger stones. The stone size in our series was larger but similar to their study, no hospital readmissions were necessary. Clear advantages of dusting include a shorter procedure time and reduced risk of ureteral trauma [10]. However, this will need to be balanced with the SFR of fragmentation and retrieval technique which seems to be superior [5, 11]. Conversely, a lower operative time and slightly higher SFR were seen with basketing technique in a pediatric study of 100 patients using semi-rigid ureteroscopy [12].

The cost of consumables such as access sheath and basket or grasper typically will increase for active stone extraction in fragmentation and retrieval technique. In addition, the study from EDGE consortium showed that dusting decreased the operating room (OR) time even for larger stones [13]. However, dusting could potentially lead to fragment regrowth over time which would negate any immediate cost savings [13].

### Role of dusting and pop-dusting in future

It seems that there will be a gradual rise in the treatment of stones using dusting and pop-dusting techniques. While dusting has been popularised with the influx of high-powered laser (100/120 W), this can be done using low-powered (20–30 W) laser too [6]. Concerning the settings, a low energy with long pulse allows smaller fragments with less retropulsion and combined with very high frequency the procedure time can be reduced. Towards the end, a pop-dusting technique can be used to finish the procedure [2].

In a recent survey of Endourology society members, 67% were using the dusting setting which reaffirms its popularity as a choice of stone treatment [14]. However, lasers that can achieve frequency of 30–70 Hz are usually expensive, which currently limits its use in most parts of the world. With increased uptake of this technique and a more widespread laser availability, this cost is potentially going to decrease, and this is unlikely to be a rate-limiting step for most endourologists.

### Strengths and limitations of the study

Our study was done in a prospective fashion for consecutive patients who had a minimum stone size of 15 mm. These were done by a single surgeon with a standard technique (published previously) eliminating any interuser variability [7]. A high-powered laser was used for the procedure as described by the previous papers [4, 6, 13]. Although the study was done prospectively, stone burden or cost analysis was not performed. Also, a head-to-head comparison with fragmentation and retrieval technique was not carried out in a previous study on stone fragmentation and retrieval for large stones achieved similar results [15].

### Areas of future research

It seems that dusting and pop-dusting not only allow faster rates of stone disintegration but also obviate the need for using UAS. Long-term follow-up on stone regrowth would determine if this ‘dust’ drains out naturally or increases the stone recurrences with time. The initial follow-up after stone dusting is also debatable and it is unclear how long it takes for the dust to completely clear out [5]. Laser fiber diameter, method of cleaving and laser technology also play a part in dusting and pop-dusting, and future studies also need to evaluate this aspect [16–18]. The impact of Moses technology in relationship to dusting and pop-dusting is still unclear and perhaps might carry an advantage due to decreased retropulsion, but this aspect needs to be studied further [19].

## Conclusion

Dusting and pop-dusting techniques achieve an excellent SFR with low risk of complications even for large stones. This might set a new benchmark for treating large stones, bilateral or multiple stones in a single setting without the need for secondary procedures in most cases.
